# Supplementary material for: Single Mutations in Cytochrome P450 Oxidoreductase Can Alter the Specificity of Human Cytochrome P450 1A2-Mediated Caffeine Metabolism
Source: Biomolecules. 2023 Jul 6;13(7):1083. doi: 10.3390/biom13071083 (PMC10377444; doi:10.3390/biom13071083)
Supplement: Supplementary file 1 [file biomolecules-13-01083-s001.zip › biomolecules-2478492-supplementary.pdf]

Supplementary Materials

Single Mutations in Cytochrome P450 Oxidoreductase can alter Specificity of Human Cytochrome P450 1A2 mediated Caffeine Metabolism

Francisco Esteves[1,*], Cristina MM Almeida[2,3], Sofia Silva[3], Inês Saldanha[1], Philippe Urban[4], José Rueff[1], Denis Pompon[4], Gilles Truan[4], Michel Kranendonk[1,*]

1 ToxOmics, NOVA Medical School, Faculdade de Ciências Médicas, NMS|FCM, Universidade NOVA de Lisboa, Campo Mártires da Pátria 130, 1169-056 Lisbon, Portugal

2 iMed.UL (Institute for Medicines and Pharmaceutical Sciences, Portugal), Faculty of Pharmacy, University of Lisboa, Av. Prof. Gama Pinto, 2, 1649-003 Lisboa, Portugal

3 Laboratory of Bromatology and Water Quality, Faculty of Pharmacy, University of Lisbon, Av. Prof. Gama Pinto, 2, 1649-003 Lisboa, Portugal

4 Toulouse Biotechnology Institute, Université de Toulouse, CNRS, INRAE, INSA, Toulouse, France

* Correspondence: FE: francisco.esteves@nms.unl.pt; MK: michel.kranendonk@nms.unl.pt

Table S1. Optimized MS/MS parameters for the chromatographic method.

Table S2. Mass spectrometer conditions used for PhCs analysis by UPLC-MS/MS.

Table S3. CPR electron donation capacity of the five FMN domain natural occurring variants.

Table S4. Caffeine consumption and ROS production rates of the BTC1A2 membrane fractions.

Figure S1. Electrostatic potential of the FD of CPR variants T142A, V164M, and H183Y.

Figure S2. Cytochrome *c* reduction activity rates of four CPR variants.

Figure S3. Detail of the interaction network between FMN and heme in the complex.

Figure S4. Number of observed residues contacts between the FD and CYP1A2 proximal side in 13 optimized complex poses.

Figure S5. Fourth orientation present in the first 12 clusters obtained after caffeine docking to CYP1A2.

Figure S6. MD simulations of the caffeine-free CYP1A2 and three caffeine-bound structures.

Figure S7. RMSF differences, for all residues of CYP1A2, between the caffeine-bound simulations and the caffeine free simulation.

Figure S8. RMSF of all atoms of CYP1A2 residues Q364, E446, F147 and P295.

Table S1. Optimized MS/MS parameters for the chromatographic method

MS/MS parameters	Acidic Method
Ionization	Electrospray
Q1 and Q3 resolution	0.7
Collision gas	Argon
Sheath, aux and sweep gas	Nitrogen
Sheath gas (Arb)	40
Aux gas (Arb)	12
CID gas (mTorr)	1.5
Vaporizer temperature (°C)	220
Ion transfer tube temperature (°C)	325

For each compound, after optimization of the triple quadrupole conditions, two transitions (product ion) were also established, one for quantification (MRM1) and one for confirmation (MRM2). The transition conditions were optimized and selected by injecting the individual standard of each compound with concentrations around 2 mg/L, in the Rheodyne valve of the MS (without chromatographic separation). The optimized mass spectrometer conditions are summarized in **Table S2**.

Stock solutions and standards for UPLC-MS/MS

Individual standard stock solutions were prepared by dissolving each target compound in reagent water (200 mg/L) and stored in glass-stoppered bottles at 5 ± 3 °C in the absence of light. For a complete dissolution of target compounds, acid formic was added at 0.4% (m/v). A working mixture solution was prepared by diluting each stock solution in reagent water to a concentration of 500 μg/L. A series of working solutions were prepared daily by diluting appropriate amounts of working mixture solution with reagent water to seven concentration levels (0.5-10 μg/L). An acceptance criterion of 0.995 and 5.0% were adopted for the coefficient of determination (R^2) and coefficient of determination of the method (CV_m), respectively. Two independent stock solutions were prepared for each pharmaceutically active compound (PhAC), one for standard solutions preparation (calibration curve) and the second one as control solutions (for trueness assay). Daily the standard error of the control solution was evaluated. An acceptance criterion of 10% was adopted for the relative error of standard solution controls.

Table S2. Mass spectrometer conditions used for PhCs analysis by UPLC-MS/MS.

Compound	Ionization mode	t_R window (individual t_R) (min)	MRM transition Precursor ion – Product ion		Collision energy (V)
TB	$[M - H]^+$	0.5 - 1.5 (0.90)	MRM1	181.1 → 163.0	18
			MRM2	181.1 → 138.0	17
PX	$[M - H]^+$	1.0 – 2.0 (1.35)	MRM1	181.3 → 123.8	19
			MRM2	181.3 → 96.0	24
TF	$[M - H]^+$	1.0 - 2.0	MRM1	181.0 → 124.0	19

		(1.6)	MRM2	181.0 → 96.0	22
CAF	[M - H]+	3.0 -7.0 (3.5)	MRM1	195.1 → 138.1	20
			MRM2	195.1 → 110.2	23

TB: theobromine; PX: paraxanthine; TF: theophylline; CAF: caffeine.

Table S3. CPR electron donation capacity of the five FMN domain natural occurring variants.

CPR Form	DCPIP reduction Initial rates (min^{-1})
**Wt**	2710 ± 251
**T142A**	2331 ± 154 *
**Q153R**	2689 ± 104
**V164M**	2772 ± 189
**D211N**	2477 ± 162
**P228L**	2596 ± 123

DCPIP reduction rates are mean ± SD (technical replicates N = 3; * $p < 0.05$).

Table S4. Caffeine consumption and ROS production rates of the BTC1A2 membrane fractions.

Membrane Fractions		Activity	
**CPR region**	**CPR Form**	**Caffeine consumption (pmol / well . min^{-1})**	**ROS production**
**FMN domain (natural variants)**	**T142A**	3.23 ± 0.10	$1.23 \times 10^{-2} \pm 1.37 \times 10^{-3}$
	**Q153R**	7.20 ± 0.37	$1.03 \times 10^{-2} \pm 7.88 \times 10^{-4}$
	**V164M**	5.64 ± 0.27	$9.11 \times 10^{-3} \pm 2.98 \times 10^{-4}$
	**D211N**	6.30 ± 0.17	$1.67 \times 10^{-3} \pm 6.32 \times 10^{-4}$
	**P228L**	5.32 ± 0.17	$2.49 \times 10^{-3} \pm 3.20 \times 10^{-6}$
**FMN domain (selected targets)**	**P117H**	5.16 ± 0.17	$4.54 \times 10^{-3} \pm 4.32 \times 10^{-5}$
	**G144C**	5.22 ± 0.06	$4.72 \times 10^{-3} \pm 1.99 \times 10^{-4}$
	**N151D**	4.92 ± 0.04	$4.83 \times 10^{-3} \pm 3.61 \times 10^{-5}$
	**G175D**	5.05 ± 0.25	$5.96 \times 10^{-3} \pm 6.75 \times 10^{-4}$
	**H183Y**	7.81 ± 0.04	$1.32 \times 10^{-2} \pm 8.94 \times 10^{-4}$
	**A229T**	7.39 ± 0.16	$1.22 \times 10^{-2} \pm 2.38 \times 10^{-3}$
**Hinge region (selected targets)**	**S243P**	5.45 ± 0.03	$1.58 \times 10^{-3} \pm 4.79 \times 10^{-4}$
	**I245P**	4.04 ± 0.05	$3.02 \times 10^{-3} \pm 2.27 \times 10^{-4}$
	**R246A**	6.61 ± 0.13	$4.53 \times 10^{-3} \pm 1.46 \times 10^{-3}$
**-**	**Wt (A)**	5.57 ± 0.16	$5.09 \times 10^{-3} \pm 5.15 \times 10^{-4}$
**-**	**Wt (B)**	5.69 ± 0.13	$1.19 \times 10^{-2} \pm 2.73 \times 10^{-3}$
**-**	**Wt (C)**	5.71 ± 0.12	$1.13 \times 10^{-2} \pm 2.42 \times 10^{-3}$
**-**	**BTC0** [a]	ND	$1.25 \times 10^{-3} \pm 2.73 \times 10^{-4}$

[a] ROS production in the reactions with the control BTC0 (membrane fraction isolated from *E. coli* cell model BTC with no expression of CPR or CYP) was determined using equal content of total protein as the one used in the Wt (B) assay. Values are mean ± SD (technical replicates N = 3).

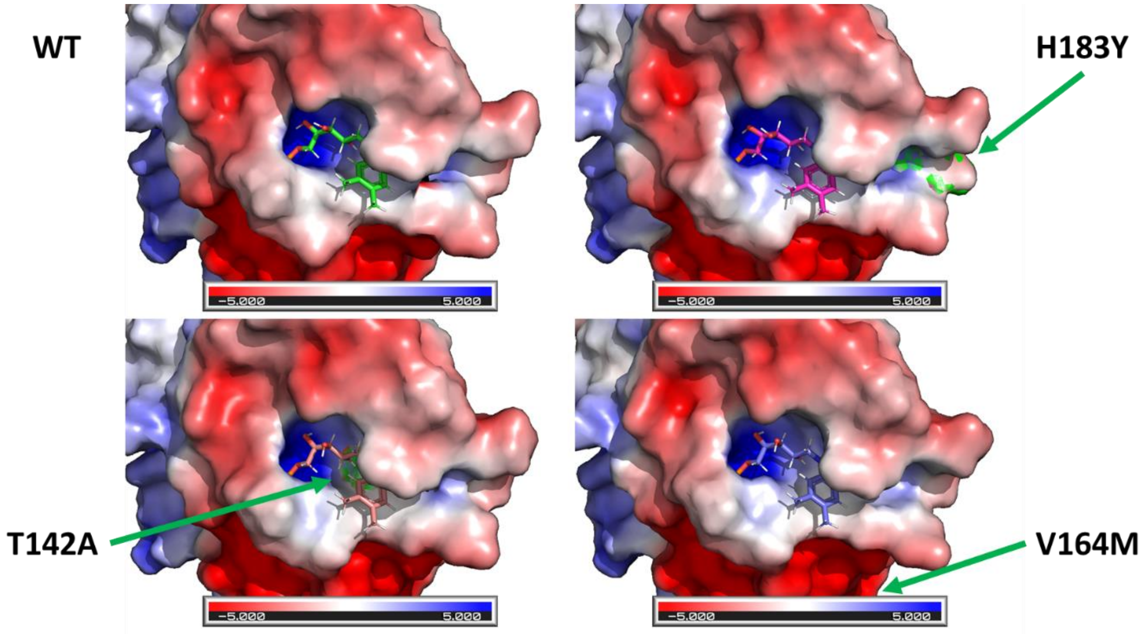

Figure S1. Electrostatic potential of the FD of CPR variants T142A, V164M, and H183Y mapped onto the Connoly surface, in comparison with CPRwt (WT). Structures of the FD mutants were obtained as described in Material and Methods. The figure was prepared with the Pymol software.

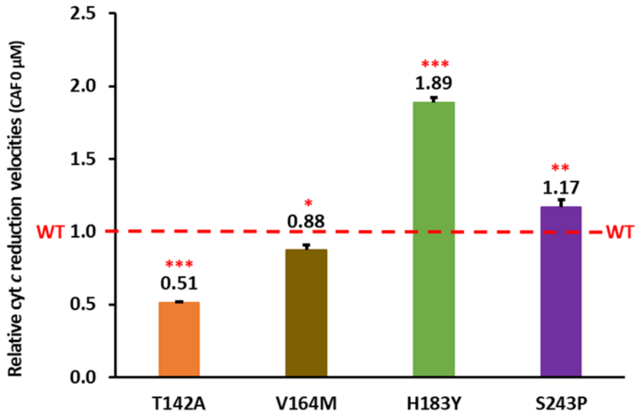

Figure S2. Cytochrome *c* reduction activity rates of four CPR variants, without caffeine. Observed rate constants of cyt *c* reduction (k_{obs}) (x fold) of the CPRvar/1A2 couples, normalized by the CPRwt activity (CAF: caffeine; WT: CPRwt/CYP1A2, red stripes) (technical replicates N = 3; *** $p < 0.001$; ** $p < 0.01$; * $p < 0.05$).

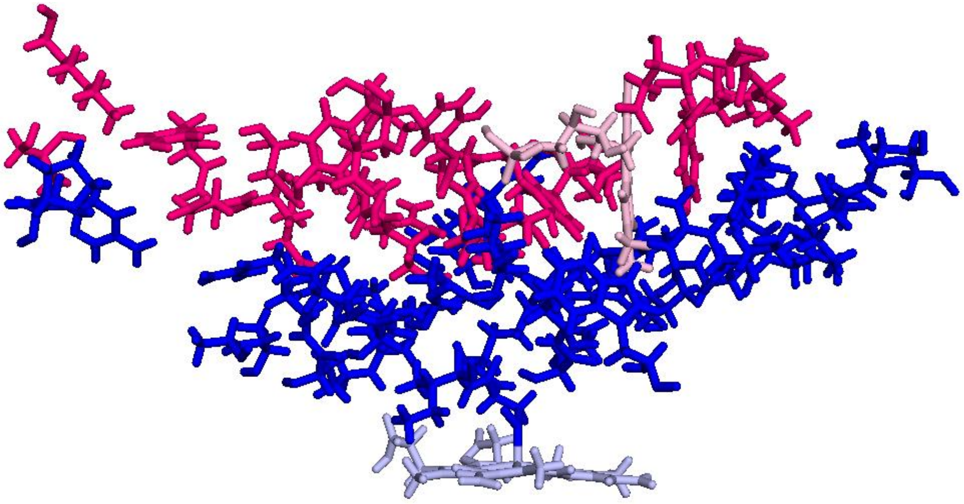

Figure S3. Detail of the interaction network between FMN (pink) and heme (light blue) in the complex. Figure is derived from the same model used for Figure 4. Side chains are depicted in blue (CYP1A2) and magenta (FD).

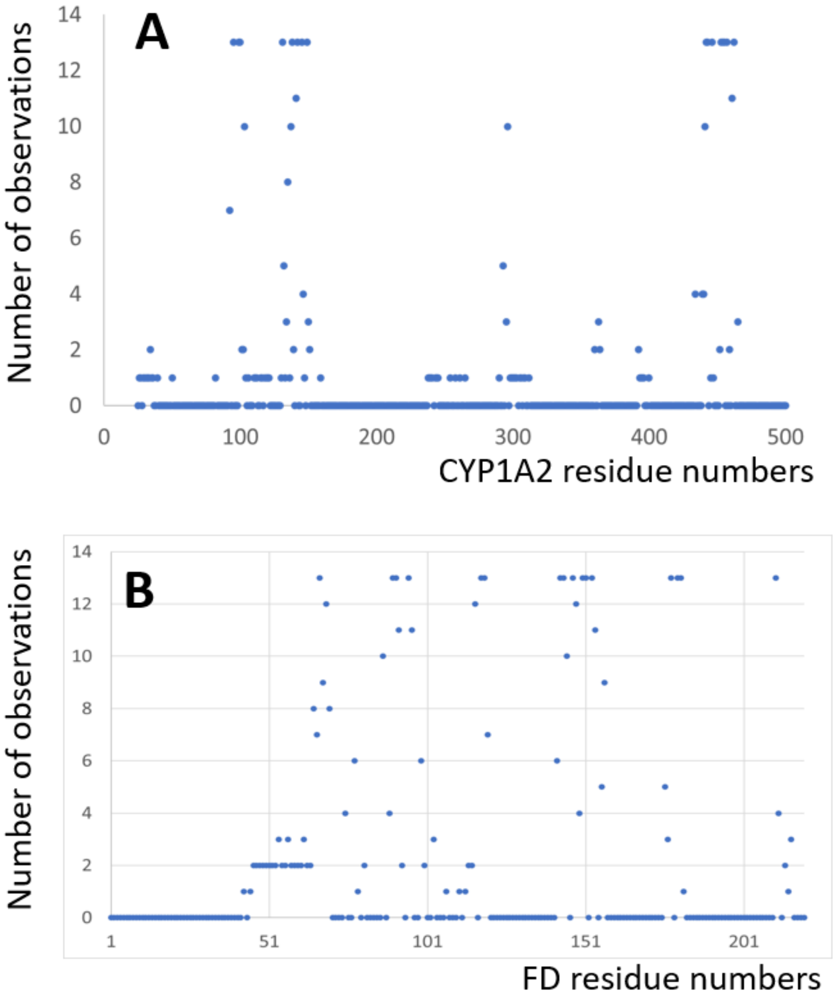

FD interface hits

Residues	Hits	Residues	Hits
67	13	54	3
90	13	57	3
91	13	62	3
95	13	103	3
118	13	177	3
119	13	216	3
143	13	46	2
144	13	47	2
147	13	48	2
150	13	49	2
151	13	50	2
153	13	51	2
178	13	52	2
180	13	53	2
181	13	55	2
211	13	56	2
69	12	58	2
116	12	59	2
148	12	60	2
92	11	61	2
96	11	63	2
154	11	64	2
87	10	81	2
145	10	93	2
68	9	100	2
157	9	114	2
65	8	115	2
70	8	214	2
66	7	43	1
120	7	45	1
78	6	79	1
99	6	107	1
142	6	111	1
156	5	113	1
176	5	182	1
75	4	215	1
89	4		
149	4		
212	4		

Figure S4. Number of observed residue contacts between the FD and CYP1A2 proximal side in 13 optimized complex poses. (A) Number of hits as function of the P450 residue number; (B) Number of hits as function of the FMN binding domain residue number. The table on the right details the B panel values above 0.

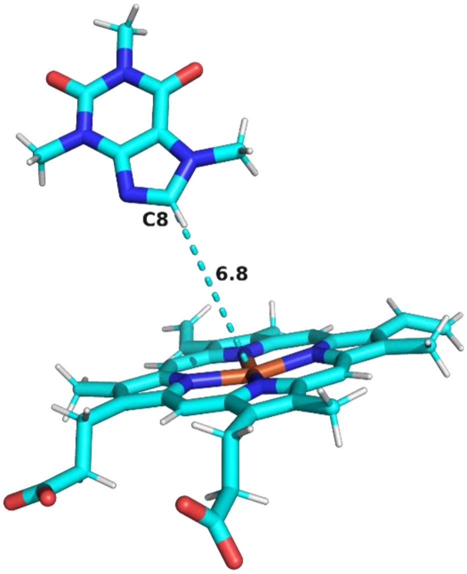

Figure S5. Fourth orientation present in the first 12 clusters obtained after caffeine docking to CYP1A2. This orientation corresponds to C8 hydroxylation.

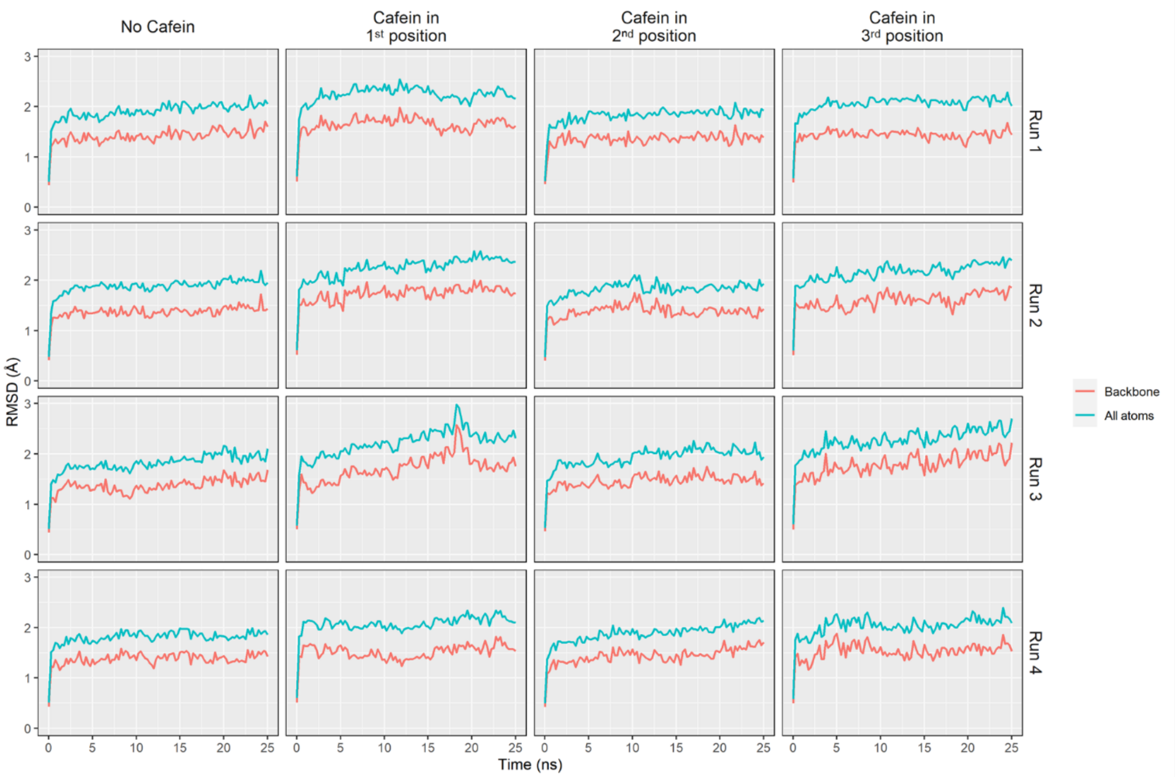

Figure S6. MD simulations (25 ns, 4 independent simulations) of the caffeine-free CYP1A2 and three caffeine-bound structures. Red, RMSD for backbone atoms, blue, RMSD for all atoms.

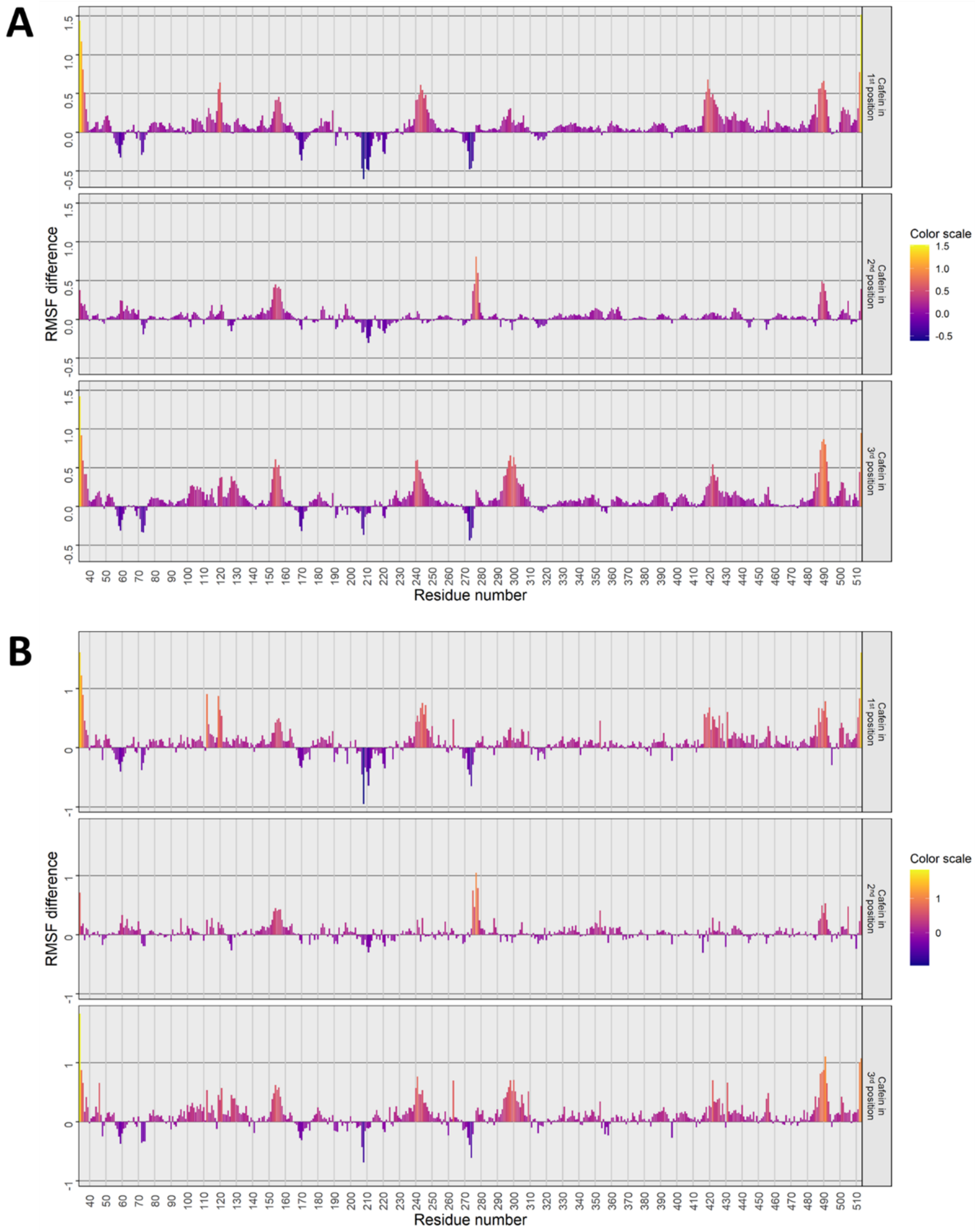

Figure S7. RMSF differences, for all residues of CYP1A2, between the caffeine-bound simulations (average of four independent runs) and the caffeine free simulation (average of four independent runs). The color scale is depicted on the right and corresponds to the difference RMSF. (A) Backbone atoms; (B) All atoms.

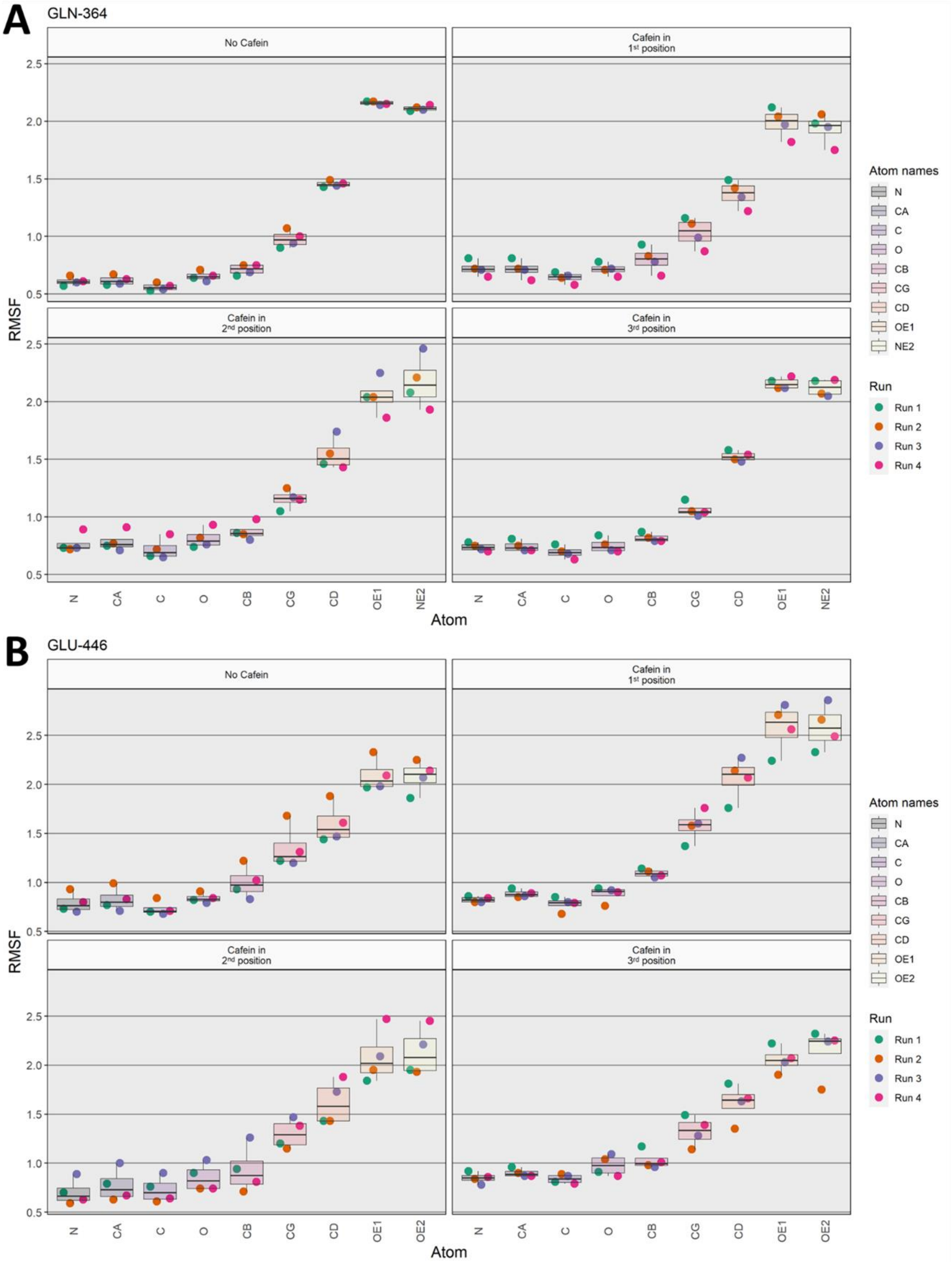
A
GLN-364
No Cafein
Cafein in 1st position
Cafein in 2nd position
Cafein in 3rd position
RMSF
0.5
1.0
1.5
2.0
2.5
N
CA
C
O
CB
CG
CD
OE1
NE2
Atom
Atom names
N
CA
C
O
CB
CG
CD
OE1
NE2
Run
Run 1
Run 2
Run 3
Run 4
B
GLU-446
No Cafein
Cafein in 1st position
Cafein in 2nd position
Cafein in 3rd position
RMSF
0.5
1.0
1.5
2.0
2.5
N
CA
C
O
CB
CG
CD
OE1
OE2
Atom
Atom names
N
CA
C
O
CB
CG
CD
OE1
OE2
Run
Run 1
Run 2
Run 3
Run 4

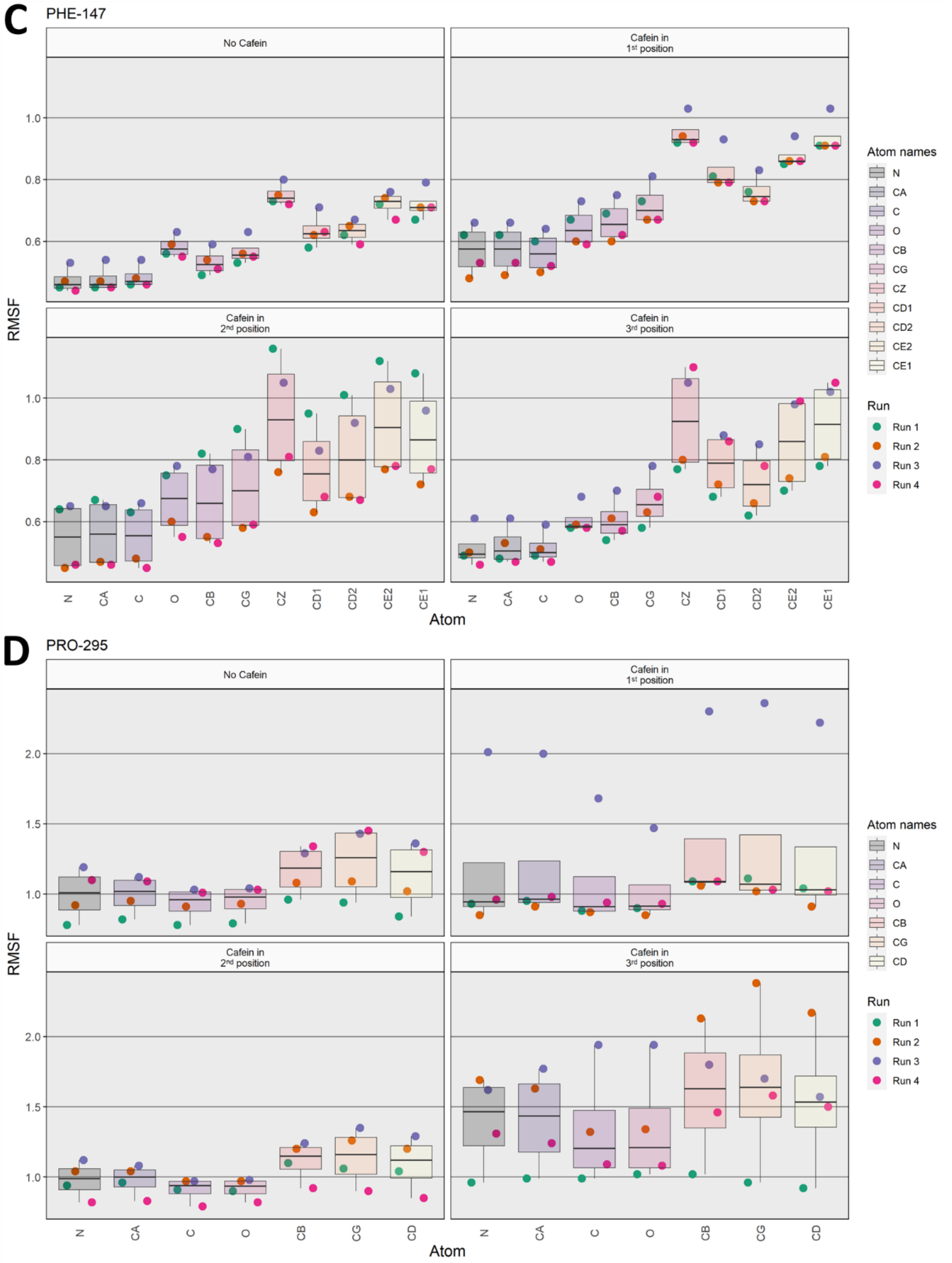

Figure S8. RMSF of all atoms of CYP1A2 residues (A) Q364, (B) E446, (C) F147 and (D) P295. All RMSF are plotted as single-colored dots using a different color scheme per molecular dynamics run. Averages, median, upper and lower limits are also depicted. The x axis corresponds to the different atoms, the y axis gives the RMSF values in angstrom.
